# Effectiveness of traditional Chinese medicine music therapy on anxiety and depression emotions of lung cancer patients: A protocol for systematic review and meta-analysis

**DOI:** 10.1097/MD.0000000000025040

**Published:** 2021-03-26

**Authors:** Xiaolin Jiang, Jing Gao, Yuping Zheng

**Affiliations:** Chengdu University of Traditional Chinese Medicine, Chengdu, Sichuan Province, China.

**Keywords:** anxiety, depression, five-element music therapy, lung cancer, meta-analysis

## Abstract

**Background:**

Lung cancer patients often accompanied with severe depression and anxiety emotions, and those negative emotions could affects the treatment and recovery of the illness, decrease the patients’ quality of life. In recent years, traditional Chinese medicine five-element music therapy (TCM-FEMT) is widely used for psychological problems of lung cancer patients for its unique advantages, TCM-FEMT applied to negative emotions management of lung cancer patients has been reported in many publications, but there is lacks evidence-based medicine, in this study, effectiveness of TCM-FEMT on anxiety and depression emotions of lung cancer patients will be systematically evaluated.

**Methods:**

PubMed, the Cochrane Library, Web of Science, Embase, Chinese Biomedical Literature Database, China National Knowledge Infrastructure, VIP Database, WanFang Database were electronically searched to collect RCTs on the efficacy of TCM-FEMT on anxiety and depression emotions of lung cancer patients from inception to February 2021. In addition, retrospect the references of the included literature to supplement the relevant literature. Research selection, data extraction and quality evaluation of literature will be carried out by 2 researchers, respectively. RevMan 5.3 software will be used for statistical analysis.

**Results:**

This study will comprehensively summarize the current trials to determine the effectiveness of TCM-FEMT on anxiety and depression emotions of lung cancer patients.

**Conclusion:**

This study will provide comprehensive evidence for the efficacy of TCM-FEMT on anxiety and depression emotions of lung cancer patients.

## 1 Introduction

According to the global cancer statistics 2018, Lung cancer is the most commonly diagnosed cancer and is the leading cause of cancer deaths worldwide.^[1]^ Most patients are already in the advanced stage when they are first diagnosed, due to the side effect of treatment, poor treatment effect and prognosis and heavy medical economic burden, patients frequently suffer from severe negative emotions such as depression and anxiety, the prevalence rates of anxiety and depression in lung cancer survivors are 34% and 33%, respectively.^[2]^ Those could affects the treatment and recovery of the illness, decrease the quality of life of patients.^[3]^ The World Health Organization has identified cancers as psychosocial diseases, in which stress is an important factor leading to negative emotions and cancers. Depression and other negative emotions could increase the incidence of cancer and promote the progression of cancer by disturbing neuroendocrine function and decreasing body's immunity.^[4,5]^ Emotion management is very important for lung cancer patients.

Music therapy, meditation, stress management, and yoga are recommended for anxiety and stress reduction. In recent years, music therapy is popular used for psychological problems of cancer patients for its positive efficacy, easily operation and high safety.^[6]^ And previous studies showed that music therapy was beneficial to the emotion regulation and symptom management of cancer patients and could help them develop a positive attitude toward the disease.^[7–9]^ Especially, traditional Chinese medicine five-element music therapy (TCM-FEMT) as the traditional Chinese music therapy, which was based on the theories of Yin-Yang and Five-element, was part of the Traditional Chinese Medicine (TCM). It is divided into five tones (Jiao, Zhi, Gong, Shang, Yu) due to the nature of the music, which connect the five internal organs (Liver, Heart, Spleen, Lung, Kidney), and the five emotions (Anger, Joy, Anxiety, Worry, Fear).^[10]^ Through balancing Yin and Yang, regulating the circulation of chi and blood in the human body, TCM-FEMT help for both physically and psychologically.^[11]^ There were studies found that TCM-FEMT could ameliorate the depression and anxiety of lung cancer patients, and ultimately improve the quality of life.^[12–14]^ But there is a lack of related systematic review to quantitatively evaluate its efficacy. Therefore, this study will to systematically review the efficacy of TCM-FEMT on anxiety and depression emotions of lung cancer patients.

## 2 Objective

To evaluate the effectiveness of TCM-FEMT on anxiety and depression emotions of lung cancer patients.

## 3 Methods

### 3.1 Protocol registration

This study has been registered on the International Platform of Registered Systematic Review and Meta-analysis Protocols (INPLASY), the registration number is INPLASY202120021, and the DOI number is 10.37766/inplasy2021.2.0021. This study was conducted under the guidance of the Preferred Reporting Items for Systematic Reviews and Meta analyses Protocols (PRISMA-P).^[15]^

### 3.2 Ethics

For this is a systematic review and meta-analysis which with no patient recruitment and any detailed personal information collection, therefore, approval from the Ethics Committee is not required.

### 3.3 Eligibility criteria

#### 3.3.1 Types of studies

All randomized controlled trials (RCTs) of TCM-FEMT on anxiety and depression emotions of lung cancer patients will be collected in this study.

#### 3.3.2 Object of study

Lung cancer patients with negative emotions of anxiety and depression. Regardless the nationality, race, body mass, and course of illness.

#### 3.3.3 Intervening measures

Five-element music therapy was used in the treatment group, while The control group included usual care, or other positive interventions groups, and there is no limitation on the intervention time, course of treatment.

#### 3.3.4 Outcome indicators

The primary outcomes assessed will be the Self-Rating Anxiety Scale (SAS), Self-Rating Depression Scale (SDS), or Hamilton Anxiety Scale (HAMA) and Hamilton Depression Scale (HAMD),^[16]^ for secondary outcome measures include the Pittsburgh Sleep Quality Index (PSQI)^[17]^ and The Short Form-36 Health Survey (SF-36).^[18]^

#### 3.3.5 Exclusion criteria

(1) Articles unrelated to the purpose of the study or nonrandomized controlled trials;
(2) Studies repeated publication;
(3) Full-text articles that cannot be retrieved through online databases, libraries, or research authors;
(4) The data is incomplete or have obvious errors.

### 3.4 Search methods

#### 3.4.1 Information sources

The following databases: PubMed, the Cochrane Library, Web of Science, Embase and Chinese databases: Chinese Biomedical Literature Database (CBM), China National Knowledge Infrastructure (CNKI), VIP Database, WanFang Database will be electronically searched to collect RCTs on the efficacy of TCM-FEMT on anxiety and depression emotions of lung cancer patients from inception to February 2021. In addition, retrospect the references of the included literature to supplement the relevant literature.

#### 3.4.2 Search strategy

The search is carried out by a combination of subject words and free words. Such terms as “Music OR Music therapy OR Five elements music OR Five-element music OR Five-tone therapy”, “Lung Neoplasms OR Lung Cancer OR Pulmonary Cancer OR Non-Small Cell Lung Cancer OR Small Cell Lung Cancer”, “Depression OR Anxiety OR Depressions OR Depressive Symptom OR Emotional Depression”, “Randomized controlled trial OR Controlled clinical trial OR Clinical Trials”. Taking PubMed as an example, the retrieval strategy is summarized in Table 1.

**Table 1:** Search strategy for the PubMed database.

Number	Search terms
#1	Music [Mesh]
#2	Music therapy [Mesh]
#3	Five elements music [Title/Abstract]
#4	Five-element music [Title/Abstract]
#5	Five-tone therapy [Title/Abstract]
#6	#1 OR #2 OR #3 OR #4 OR #5
#7	Lung Neoplasms [Mesh]
#8	Lung Cancer [Title/Abstract]
#9	Pulmonary Cancer [Title/Abstract]
#10	Non-Small Cell Lung Cancer [Title/Abstract]
#11	Small Cell Lung Cancer [Title/Abstract]
#12	#7 OR #8 OR #9 OR #10 OR #11
#13	Depression [Mesh]
#14	Anxiety [Mesh]
#15	Depressions [Title/Abstract]
#16	Depressive Symptom [Title/Abstract]
#17	Emotional Depression [Title/Abstract]
#18	#13 OR #14 OR #15 OR #16 OR #17
#19	Randomized controlled trial [Title/Abstract]
#20	Controlled clinical trial [Title/Abstract]
#21	Clinical Trials [Title/Abstract]
#22	#19 OR #20 OR #21
#23	#6 AND #12 AND #18 AND #22

### 3.5 Data screening and extraction

**Figure 1:**
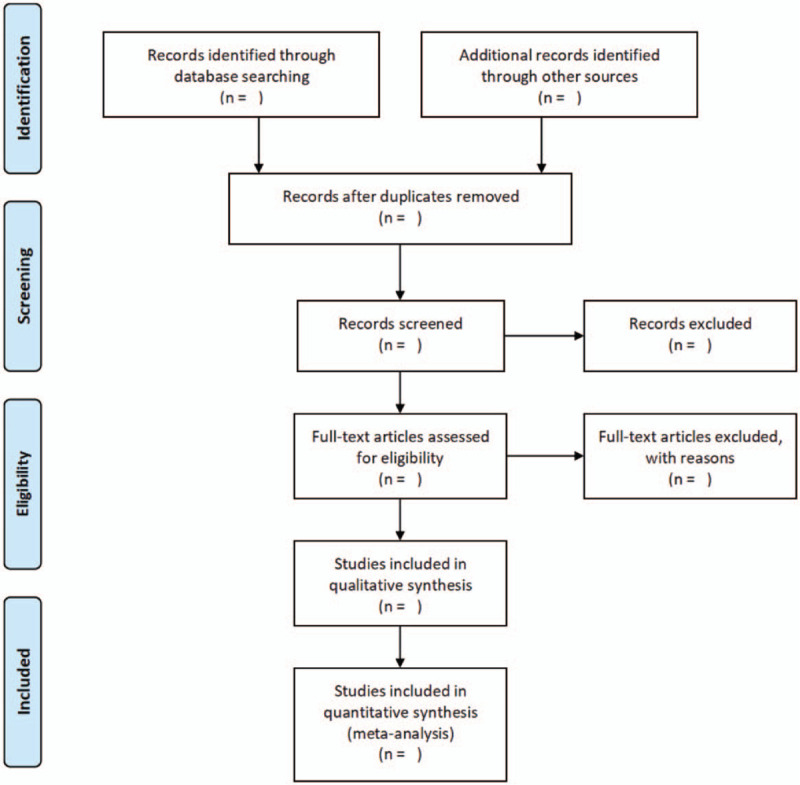
Flow diagram of study selection process.

All retrieved studies will be imported into Endnote X9 and grouped by different databases for management. By way of a combination of artificial and endnote to eliminate duplicate documents. According to eligibility criteria, data extraction will be completed by 2 researchers, independently. The literature was screened by reading the title and abstract to remove the obviously irrelevant literature, and then screened it again after reading the full text to determine whether to include. If there is a disagreement, it will be resolved by a third researcher, for lacked information, we will contact the correspondent authors via email or phone for completed the data. The following information will be extracted from the literature and recorded in a unified table: (1) Basic information of the included research: research title, first author, year of publication, journal; (2) Baseline characteristics (age, gender, sample size) and intervention measures of the research object (treatment time, course of treatment, combination therapy, follow-up time points); (3) Methodological information of literature; (4) Outcome indicators. The literature screening process is shown in Figure 1.

### 3.6 Quality assessment

RCT's risk of bias evaluation tool in Cochrane System Review Manual 5.1.0 will be used to evaluate the risk of bias for the included RCTs,^[19]^ Two researchers will independently evaluate the literature quality and cross-check the results. If there are any differences, a third researcher will participate in the discussion to make decisions.

### 3.7 Statistical analysis

#### 3.7.1 Data analysis and processing

RevMan 5.3 software will be used for statistical analysis. The measurement data uses mean difference (MD) as the effect analysis statistic, and each effect size provides its 95% confidence intervals (CIs). The heterogeneity among the included results will be analyzed using *χ*^2^ test, and the heterogeneity will quantitatively determined by combining with *I*^2^. When there is significant statistical heterogeneity among the studies (*P* < .1, *I*^2^ > 50%), the combined effect size of the random effect model was adopted, Otherwise, the fixed effect model be adopted. When the measurement tools and units are inconsistent or the mean values differ greatly of different researches, the standardized mean difference and 95% CIs will used as the composite statistics.

#### 3.7.2 Subgroup analysis

If there is heterogeneity, we will conduct a subgroup analysis, we will take an analysis of subgroup according to characteristic of participants, such as gender, age, course of disease.

#### 3.7.3 Sensibility analysis

To assess the stability of meta-analysis results, sensitivity analysis will be performed by deleting the included studies one by one to assess the change in the overall effect after removing a certain study.

#### 3.7.4 Publication bias

Egger test, Begger test and funnel plots are used to assess the potential publication bias if more than 10 studies are included in this study.^[20]^ If there is publication bias, we will use trimming and filling methods to further evaluate the impact of the deviations on the results.^[21]^

#### 3.7.5 Evidence quality

According to the Grading of Recommendations Assessment, Development and Evaluation (GRADE) (Version 3.6), The quality of evidence will be ranked as 4 levels: high, moderate, low, and very low based on 5 key domains: risk of bias, consistency, directness, precision, and publication bias.

## 4 Discussion

More and more researches used music therapy into the intervention of negative emotions in lung cancer, music as a product of human mental activity, is the manifestation of the human's inner world and the material world. The effects of music therapy has a certain connection with the body's physiology and psychology. The frequency and sound pressure of music can cause a physiological response of the body. The frequency, rhythm, and regular sound wave vibration of music belong to physical energy. When kept in a moderate range, it will cause harmonious resonance of histiocytes, resulting in resonance in the cranial cavity, thoracic cavity, or tissue, and affect brain waves, breathing rhythm, heart rate, etc. In psychology, the frequency and sound pressure of music can eliminate the patient's tension, anxiety, depression and other emotions, and improve stress tolerance.^[22]^ With the guidance of holism concept in TCM, TCM-FEMT emphasizes the unity of body and spirit, the harmonious unity of human and nature, and human and society, which consistent with the “biological-psychological-social model” proposed in modern Western medicine, it plays an increasingly important role in treating, preventing diseases, and improving the quality of life.

At present, many trials have shown the efficacy of TCM-FEMT in the treatment of anxiety and depression emotions of lung cancer patients, but there is no relevant systematic evaluation. As this is a systematic evaluation and meta-analysis to provide reliable evidence for clinical promotion of TCM-FEMT for lung cancers.

## Author contributions

**Conceptualization:** Xiaolin Jiang, Jing Gao.

**Data curation:** Xiaolin Jiang, Jing Gao, Yuping Zheng.

**Formal analysis:** Xiaolin Jiang, Yuping Zheng.

**Funding acquisition:** Jing Gao.

**Methodology:** Xiaolin Jiang, Jing Gao, Yuping Zheng.

**Project administration:** Xiaolin Jiang, Jing Gao, Yuping Zheng.

**Software:** Xiaolin Jiang, Yuping Zheng.

**Supervision:** Jing Gao.

**Writing – original draft:** Xiaolin Jiang.

**Writing – review & editing:** Xiaolin Jiang, Jing Gao.

## References

[1] Bray F Ferlay J Soerjomataram I. Global cancer statistics 2018: GLOBOCAN estimates of incidence and mortality worldwide for 36 cancers in 185 countries. CA Cancer J Clin 2018;68:394–424. 30207593 10.3322/caac.21492

[2] Krebber AM Buffart LM Kleijn G. Prevalence of depression in cancer patients: a meta-analysis of diagnostic interviews and self-report instruments. Psychooncology 2014;23:121–30. 24105788 10.1002/pon.3409 PMC4282549

[3] Sullivan DR Forsberg CW Ganzini L. Longitudinal changes in depression symptoms and survival among patients with lung cancer: a national cohort assessment. J Clin Oncol 2016;34:3984–91. 27996350 10.1200/JCO.2016.66.8459 PMC5477833

[4] Miller AH Ancoli-Israel S Bower JE. Neuroendocrine-immune mechanisms of behavioral comorbidities in patients with cancer. J Clin Oncol 2008;26:971–82. 18281672 10.1200/JCO.2007.10.7805 PMC2770012

[5] Tashiro M Itoh M Kubota K. Relationship between trait anxiety, brain activity and natural killer cell activity in cancer patients: a preliminary PET study. Psychooncology 2001;10:541–6. 11747066 10.1002/pon.548

[6] Jasemi M Aazami S Zabihi RE. The effects of music therapy on anxiety and depression of cancer patients. Indian J Palliat Care 2016;22:455–8. 27803568 10.4103/0973-1075.191823 PMC5072238

[7] Moore KS. A systematic review on the neural effects of music on emotion regulation: implications for music therapy practice. J Music Ther 2013;50:198–242. 24568004 10.1093/jmt/50.3.198

[8] Koelsch S Boehlig A Hohenadel M. The impact of acute stress on hormones and cytokines, and how their recovery is affected by music-evoked positive mood. Sci Rep 2016;29:23008. 10.1038/srep23008 PMC4810374 27020850

[9] Potvin N Bradt J Kesslick A. Expanding perspective on music therapy for symptom management in cancer care. J Music Ther 2015;52:135–67. 25755121 10.1093/jmt/thu056

[10] Zhang ZX Cai ZX Yu YH. Effect of Lixujieyu recipe in combination with five elements music therapy on chronic fatigue syndrome. J Tradit Chin Med 2015;35:637–41. 26742307 10.1016/s0254-6272(15)30152-7

[11] Chen CJ Sung HC Lee MS. The effects of Chinese five-element music therapy on nursing students with depressed mood. Int J Nurs Pract 2015;21:192–9. 24593291 10.1111/ijn.12236

[12] Juan L Yang YF Cohen L. Effects of Chinese medicine five-element music on the quality of life for advanced cancer patients: a randomized controlled trial. Chin J Integr Med 2013;9:736–40. 10.1007/s11655-013-1593-5 24092240

[13] Sun FY Zhou Q. Effect of five elements music intervention on negative emotions in patients with intravenous chemotherapy of lung cancer. J Nurs Compr Ed (Chin) 2015;30:35–7.

[14] Wang M Chen FR Jiang MY. Effect of music on quality of life in patients with anxiety and depression lung cancer during chemotherapy. Nurs Res 2011;25:2478–9.

[15] Shamseer L Moher D Clarke M. Preferred reporting items for systematic review and meta-analysis protocols (PRISMA-P) 2015: elaboration and explanation. BMJ 2015;350:g7647. 25555855 10.1136/bmj.g7647

[16] Wang XD Wang XL Ma H. Manual of Mental Health Rating Scale. Revised Ed 1999;Beijing: Chinese Journal of Mental Health, 31–35.

[17] Buysse DJ Reynolds CF 3rd Monk TH. The Pittsburgh Sleep Quality Index: a new instrument for psychiatric practice and research. Psychiatry Res 1989;28:193–213. 2748771 10.1016/0165-1781(89)90047-4

[18] Li L Wang HM Shen Y. Evelopment and psychometric tests of a Chinese version of the SF-36 Health Survey Scales. Chin J Prev Med 2002;2:38–42. 12410965

[19] Higgins JPT Green S. Cochrane handbook for systematic reviews of interventions (Version 5.1.0). The Cochrane Collaboration 2011. Available at: http://www.cochrane-handbook.org.

[20] Egger M Smith GD Phillips AN. Meta-analysis: principles and procedures. BMJ 1997;315:1533–7. 9432252 10.1136/bmj.315.7121.1533 PMC2127925

[21] Duval S Tweedie R. Trim and fill: a simple funnel-plot-based method of testing and adjusting for publication bias in meta-analysis. Biometrics 2000;56:455–63. 10877304 10.1111/j.0006-341x.2000.00455.x

[22] Bradt J Dileo C Magill L. Music interventions for improving psychological and physical outcomes in cancer patients. Cochrane Database Syst Rev 2016;8:CD006911. 10.1002/14651858.CD006911.pub3 27524661

